# *Bacillus thuringiensis* and Silicon Modulate Antioxidant Metabolism and Improve the Physiological Traits to Confer Salt Tolerance in Lettuce

**DOI:** 10.3390/plants10051025

**Published:** 2021-05-20

**Authors:** Muneera ALKahtani, Yaser Hafez, Kotb Attia, Talal Al-Ateeq, Mohamed A. M. Ali, Mirza Hasanuzzaman, Khaled Abdelaal

**Affiliations:** 1 Biology Department, College of Science, Princess Nourah Bint Abdulrahman University, Riyadh POX 102275-11675, Saudi Arabia; 2 Excellence Center (EPCRS), Plant Pathology and Biotechnology Lab, Faculty of Agriculture, Kafrelsheikh University, Kafr El-Sheikh 33516, Egypt; hafezyasser@gmail.com; 3 Center of Excellence in Biotechnology Research, King Saud University, Riyadh POX 2455-11451, Saudi Arabia; ksmattia@yahoo.com (K.A.); talalatiq@gmail.com (T.A.-A.); 4 Rice Biotechnology Lab, Rice Department, Field Crops Research Institute, ARC, Sakha 33717, Egypt; 5 Department of Horticulture, Faculty of Agriculture, New Valley University, El-Kharga 72511, Egypt; mohamed.ali@agr.nvu.edu.eg; 6 Department of Agronomy, Faculty of Agriculture, Sher-e-Bangla Agricultural University, Dhaka 1207, Bangladesh; mhzsauag@yahoo.com

**Keywords:** silicon, lettuce, salt stress, *Bacillus*, antioxidant enzymes activity, yield

## Abstract

We investigated the impact of *Bacillus thuringiensis* as seed treatment and application with silicon on lettuce plants exposed to salinity levels (4 dS m^−1^ and 8 dS m^−1^). Results revealed that leaves number, head weight, total yield, relative water content (RWC), and chlorophyll a and b declined considerably due to two salinity levels. Oxidative stress markers, i.e., hydrogen peroxide (H_2_O_2_), superoxide (O_2_^−^), and lipid peroxidation (MDA) dramatically augmented in stressed plants. On the other hand, leaves number, total yield, RWC, and chlorophyll a, b in stressed lettuce plants were considerably enhanced because of the application of Si or *B. thuringiensis.* In contrast, EL%, MDA, and H_2_O_2_ were considerably reduced in treated lettuce plants with Si and *B. thuringiensis*. In addition, the treatment with Si and *B. thuringiensis* increased head weight (g) and total yield (ton hectare-1), and caused up-regulation of proline and catalase, superoxide dismutase, peroxidase, and polyphenol oxidase activity in lettuce leaves under salinity conditions.

## 1. Introduction

Lettuce plants (*Lactuca sativa* L.) belong to the Asteraceae family. It is an important and popular vegetable crop grown as an annual plant, which is used as a salad crop. It is a rich source of vitamins and antioxidants such as vitamins C, carotenoids, and fiber content [1]. Some studies suggest that lettuce may have originated in the Mediterranean region [2], it is a very important commercial crop in North and Central America, Asia, Europe, and Egypt [3]. Japan, China, the U.S.A, Spain, Italy, and India are among the largest producers [4,5]. Environmental stress factors such as salinity [6,7,8,9], drought [10,11,12,13,14,15,16], chilling, and heat stress [17,18] negatively affect crop productivity in many plants. Salinity is an abiotic harmful stress factor, affecting structure, physiochemical characters, and the ecology of the soil, as well as the growth and plant yield [19,20,21]. The total area of saline and sodic soils was determined (831 million ha) worldwide according to the FAO report [22]. The salt-affected soils were divided into two main groups according to their physical, biological, and chemical properties [23]. The first group is saline soil, which contains large quantities of neutral soluble salts, mainly sodium chloride and sodium sulphate, and also contains considerable quantities of calcium chloride and magnesium chloride. However, the second group is sodic soil, which contains sodium salts capable of alkaline hydrolysis, mostly Na_2_CO_3_. The accumulation of Na^+^ and Cl^−^ ions in the soil at higher concentrations led to increased salt concentration in soil [24]. All of the growth and developmental phases are negatively affected by salinity, such as the germination of seeds, vegetative phase, and flowering phases due to biochemical and physiological changes in plants in the form the accumulation of solutes at higher concentrations, which, negatively affect K^+^/ Na^+^ ratio and nutrient status resulting in oxidative damage [25,26]. Under salinity conditions, chlorophyll concentration, electrolyte leakage, and RWC are significantly reduced in calendula plants [27], and salinity led to decline leaf area and number of leaves [7], and led to increased Na^+^ accumulation, reducing the uptake of nutrients such as nitrogen and potassium [28]. Moreover, salinity is generally associated with oxidative damage due to ROS accumulation [29] such as super oxide and H_2_O_2_ which causes lipid peroxidation in numerous plants under various stress factors [30,31,32,33,34,35].

Silicon (Si) is a significant element, covering around 28% of the lithosphere, and lately it has become known as a ‘quasi-essential’ element according to The International Plant Nutrition Institute [36]. In grasses, the effects of water availability on the silicification process is stronger than in non-grass species [37]. Si led to improvements in the growth traits and yield production, particularly under stress conditions [36,38]. Si also led to mitigating the damaging impact of drought in barley plants [10], faba bean plants [11], and in sweet pepper due to salinity [36]. Also, silicon may ameliorate the environmental stresses in the context of legume–rhizobia relationships, resulting in increased resistance against pathogens and insect antagonists [39].

The application of Si mitigates the injurious impact of salinity on sweet pepper resulting in the improvement of morphophysiological characteristics, for example, number of leaves and chlorophyll (Chl) concentrations, as well as fruits yield [36]. Si can play a major role in the improvement of plant status through regulating transpiration rates resulting in improving the photosynthetic rate. Additionally, Si improves the assimilation rate of carbon dioxide under salinity in sorghum, and also Si alleviates the harmful impacts of salinity on stomatal conductance of sweet pepper and boosted zinc finger protein expression which, regulates stomatal movement in the salt stressed rice plants [40,41,42,43].

Plant growth-promoting rhizobacteria (PGPR) such as Rhizobium, Azotobacter, Bacillus, and Serratia can be used to increase plant yield under stress and normal conditions by the production of vitamins, antioxidants, and many phytohormones [44,45]. PGPR led to improved sweet pepper growth characters and fruit yield under salinity stress [6], additionally, PGPR can use as biofertilizer [46,47,48,49] and as a biocontrol agent [50]. Application of PGPR can alleviate the damaging impacts of stress and improve the yield production under stress conditions [51]. Si is an important element in agricultural sustainable production, mainly, under salinity conditions. Also, PGPR is a very important method for improving growth characters under normal and stress conditions. Accordingly, the aim of our investigation was to study the impact of Bacillus thuringiensis and Si on stimulating salt tolerance in lettuce plants associated with Chl, RWC, MDA, up-regulation of enzymes activity, proline contents, and ROS, as well as head yield in lettuce plants under salinity.

## 2. Results

### 2.1. Lipid Peroxidation (MDA), Reactive Oxygen Species (O_2_^−^ and H_2_O_2_) and Electrolyte Leakage (EL%)

**Figure 1:**
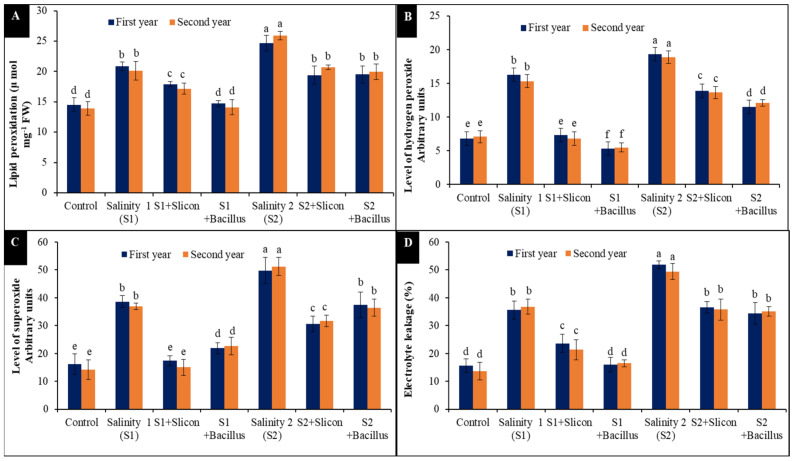
Effect of Si (potassium silicate at 2.7 mmol L^−1^) and *B. thuringiensis* on lipid peroxidation (MDA) (**A**), H_2_O_2_ (**B**), O_2_^−^ (**C**), and EL (**D**) in lettuce plants under salinity during two seasons (2019/2020 and 2020/2021). Different letters on the columns show significant differences between the treatments according to ANOVA, Duncan’s multiple range test at 0.05 level. Data is the mean (±SE) of tree replicates. Salinity 1 (S1): 4 dS m^−1^, Salinity 2 (S2): 8 dS m^−1^.

It was observed that lettuce plants exposed to salinity stress showed a considerable increase in MDA (pFW9 and Figure 1A). Plants treated with low (S1) and high (S2) levels of salinity resulted in 44% and 70% increases in MDA content compared with the control in the first year, and in the second year the increases were 44% and 87%, respectively. Conversely, MDA significantly declined in the stressed lettuce plants treated with *B. thuringiensis* and Si treatment. Si application significantly decreased MDA content in lettuce at a low salinity level (14% and 15%) compared with untreated stressed plants at a low level of salinity, 4 dS m^−1^. Also, MDA decreased significantly with Si treatment at a high level of salinity 8 dS m^−1^ (S2; 21% and 20%) compared with untreated stressed plants at high level. *B. thuringiensis* led to decreased MDA and gave the best results in MDA content (29% and 30%) at the low level of salinity and (21% and 23%) at the high level, compared to the untreated plants at low salinity levels in both seasons.

A remarkable increase in super oxide (O_2_^−^) and hydrogen peroxide (H_2_O_2_) formation was observed in the exposure lettuce plants to both salinity levels. H_2_O_2_ was significantly augmented in stressed lettuce plants (Figure 1B), the high increase was recorded at a high salinity level (183.2% and 166.19%) in comparison with control in both seasons, respectively. Conversely, H_2_O_2_ declined significantly in stressed lettuce plants, consistent with Si and *B. thuringiensis*. *B. thuringiensis* led to reduced H_2_O_2_ and gave the best results (67.42% and 38.59%) at a low salinity level and (40.50% and 35.97%) at a high level in comparison to untreated stressed plants in both seasons, respectively.

Additionally, the obtained results in Figure 1C showed that O_2_^−^ dramatically increased in the stressed lettuce plants at a low level of salinity (138.6% and 160.59%) and at a high level (208.16% and 261.79%) compared with control in both seasons, respectively. A significant decrease in O_2_^−^ was observed according to Si and *B. thuringiensis* treatments, silicon application led to a decrease in the level of O_2_^−^ at a high salinity level (38.49% and 38.2%) in comparison to untreated plants, and also *B. thuringiensis* treatment led to decreases in the level of O_2_^−^ with a low salinity level (24.69% and 28.94%) compared with untreated stressed plants in both seasons, respectively.

It is evident from the recorded results in Figure 1D that EL% considerably augmented in the lettuce stressed plants; the low salinity level led to a significant increase (56.14 and 63.04%) compared to control plants in both seasons. Moreover, the high level of salinity was more deleteriously effective on EL% (225.75% and 261.85%) compared with control treatment. However, Si application significantly declined EL% in the stressed lettuce plants at the low level (33.73% and 42.11%) and the high level (29.5% and 27.67%). Additionally, *B. thuringiensis* had a helpful effect and reduced EL% (55.13% and 55.27%) in the stressed plants with low salinity level in both seasons. Moreover, *B. thuringiensis* significantly declined EL% in the stressed treated plants at high salinity level (33.82% and 28.92%) compared with lettuce stressed untreated plants at high salinity level in both seasons.

### 2.2. The Activity of Catalase (CAT), Superoxide Dismutase (SOD), Peroxidase (POX) and Polyphenol Oxidase (PPO)

**Figure 2:**
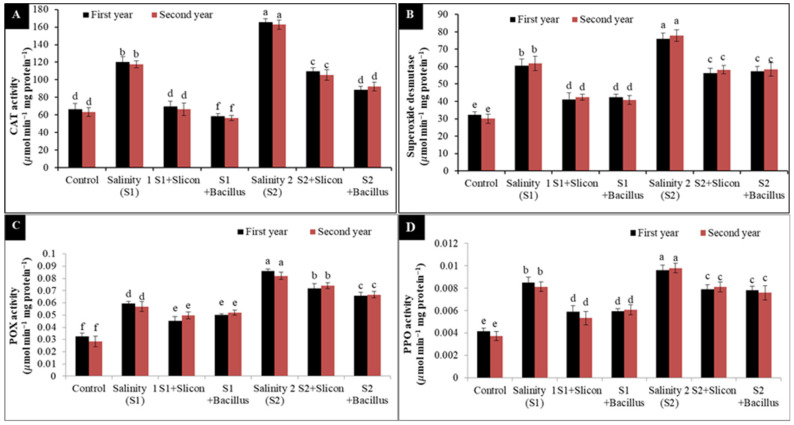
Effect of Si (potassium silicate at 2.7 mmol L^−1^) and *B. thuringiensis* on the activity of CAT (**A**), SOD (**B**), POX (**C**), and PPO (**D**) in lettuce plants under salinity during two seasons (2019/2020 and 2020/2021). Different letters on the columns show significant differences between the treatments according to ANOVA, Duncan’s multiple range test at 0.05 level. Data is the mean (±SE) of tree replicates. Salinity 1 (S1): 4 dS m^−1^, Salinity 2 (S2): 8 dS m^−1^.

CAT, SOD POX, and PPO activity was dramatically augmented in lettuce plants at both salinity levels (Figure 2A–D). CAT activity significantly increased at a low salinity level (86.91% and 88.9%) and at high salinity level (158.09% and 161.57%) in comparison with control plants in the two seasons. Nevertheless, the application of Si and *B. thuringiensis* showed positive impact on lettuce stressed plants and minimize CAT activity. *B. thuringiensis* gave a positive response and led to adjusted CAT activity at a low salinity level (102.36% and 105.06%) compared to untreated stressed plants and at a high level of salinity (87.44% and 74.19%) compared with untreated plants in both seasons. Additionally, the treatment with Si and *B. thuringiensis* led to regulated SOD activity; *B. thuringiensis* achieved 28.90% and 32.83% at a low salinity level compared to untreated stressed plants and (25.72% and 24.83%) at a high level of salinity compared with stressed untreated plants in both seasons (Figure 2B). Interestingly enough, POX and PPO activity was significantly elevated under salinity levels compared with control treatment in lettuce plants in both seasons (Figure 2C,D). However, Si and *B. thuringiensis* observed helpful impact on lettuce stressed plants and justified POX and PPO activities. *B. thuringiensis* gave a positive response and led to regulate POX and PPO activities at two levels compared to untreated stressed plants.

### 2.3. Relative Water Content (RWC%) and Proline Content

**Figure 3:**
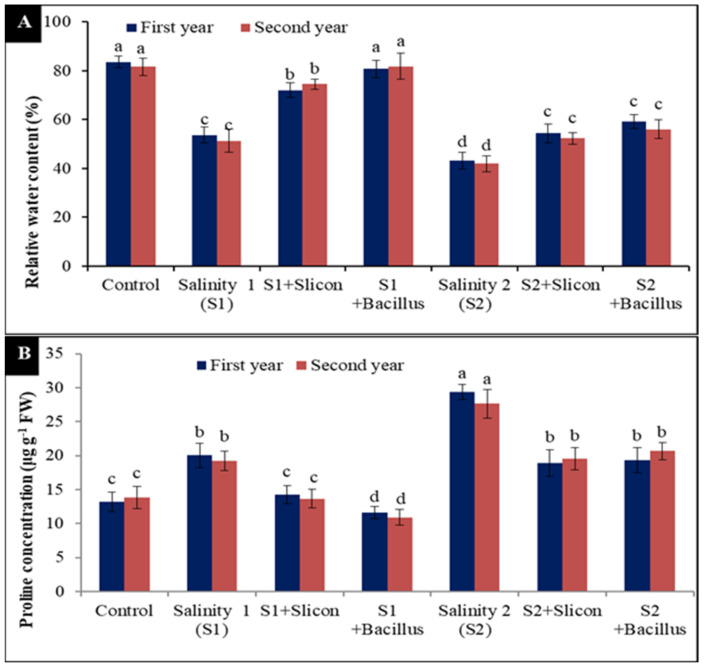
Effect of Si (potassium silicate at 2.7 mmol L^−1^) and *B. thuringiensis* on RWC (**A**) and proline content (**B**) in lettuce plants under salinity during two seasons (2019/2020 and 2020/2021). Different letters on the columns show significant differences between the treatments according to ANOVA, Duncan’s multiple range test at 0.05 level. Data is the mean (±SE) of tree replicates. Salinity 1 (S1): 4 dS m^−1^, Salinity 2 (S2): 8 dS m^−1^.

The data in Figure 3A exhibited that RWC in lettuce plants was significantly declined under both levels of salinity, the low level led to significant reductions (36.96% and 37.18%) compared with control plants in the two seasons. Meanwhile, the decrease was higher under a high level of salinity (48.32% and 48.65%) compared with control treatments in both seasons. Contrariwise, Si application considerably increased RWC in stressed lettuce plants at the low level (28.78% and 33.93%) compared with untreated stressed plants in the two seasons, respectively. Furthermore, RWC was significantly increased with *B. thuringiensis* application and recorded the best results with a low salinity level (61.4% and 67.4%) and under a high salinity level (37.16% and 33.79%) in comparison with stressed untreated plants and the control treatment.

It is evident from the recorded results in Figure 3B that, proline content significantly augmented in lettuce plants under two salinity levels, the high level gave the high content of proline in lettuce plants (123.66% and 100%) compared to the control treatment in both seasons, respectively. Conversely, the application of Si led to the regulation and diminishing of the accumulation of proline in stressed plants with a low concentration of salinity (40.48% and 41.17%) compared with stressed untreated plants in both seasons; the greatest effect on proline content was achieved with *B. thuringiensis* as seed treatment (72.41% and 76.14%) in comparison with stressed untreated plants in the two seasons.

### 2.4. Ascorbic Acid (AsA)

**Figure 4:**
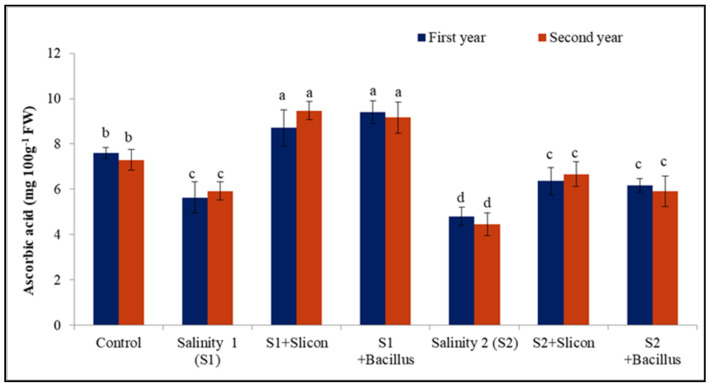
Effect of Si (potassium silicate at 2.7 mmol L^−1^) and *B. thuringiensis* on Acorbic acid in lettuce plants under salinity during two seasons (2019/2020 and 2020/2021). Different letters on the columns show significant differences between the treatments according to ANOVA, Duncan’s multiple range test at 0.05 level. Data is the mean (±SE) of tree replicates. Salinity 1 (S1): 4 dS m^−1^ Salinity 2 (S2): 8 dS m^−1^.

The results presented in Figure 4 indicated a significant reduction in ascorbic acid (AsA) in lettuce under two salinity levels (35.71%, 51.39%, and 22.03%, 63.63%) in comparison with the control in both seasons, respectively. Conversely, application of Si and *B. thuringiensis* displayed a significant increase in ascorbic acid in salt-stressed lettuce plants. Si led to a significant increase in ascorbic acid in the stressed plants with salinity levels (54.52%, 59.32% and 31.25%, 52.27%) in the two seasons. Likewise, *B. thuringiensis* gave the maximum results of AsA particularly, with the low salinity level (67.85% and 55.25%) in both seasons, respectively, in comparison with untreated stressed plants and control also.

### 2.5. Chlorophyll A and B Concentration

**Figure 5:**
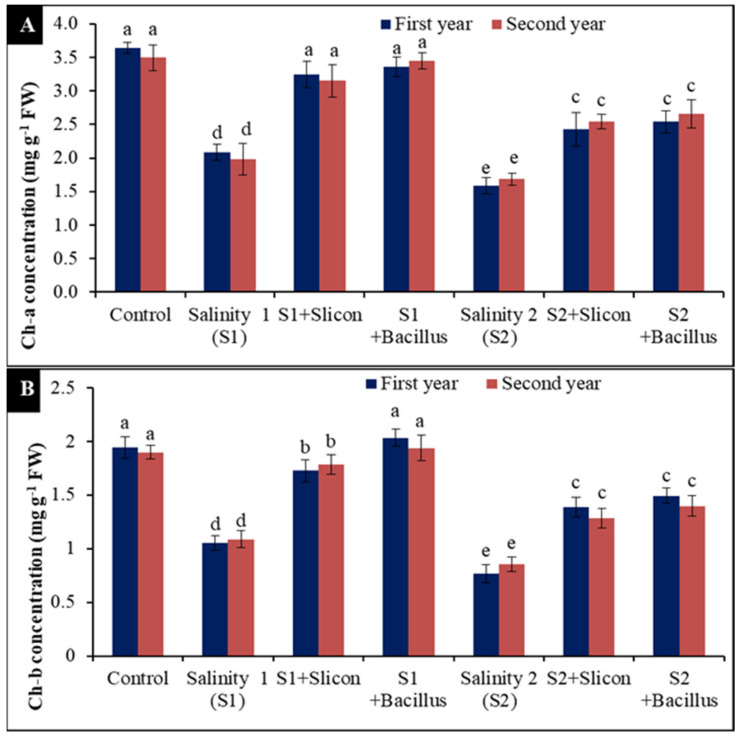
Effect of Si (potassium silicate at 2.7 mmol L^−1^) and *B. thuringiensis* on chlorophyll a (**A**) and chlorophyll b (**B**) in lettuce plants under salinity during two seasons (2019/2020 and 2020/2021). Different letters on the columns show significant differences between the treatments according to ANOVA, Duncan’s multiple range test at 0.05 level. Data is the mean (±SE) of tree replicates. Salinity 1 (S1): 4 dS m^−1^, Salinity 2 (S2): 8 dS m^−1^.

Lettuce plants grown at the different salinity levels had significantly reduced chlorophyll a and b concentrations (Figure 5A,B). A low salinity level led to significant reduction in chlorophyll a (41.66% and 42.85%) compared with the control in both seasons, while the decrease in chlorophyll a was greater under a high salinity level (55.6% and 105.7%) (Figure 5A). Application of Si and *B. thuringiensis* induced the increase in chlorophyll a concentration, *B. thuringiensis* gave the maximum concentration of chlorophyll a in the stressed plants with low salinity level (70% and 84.21%) followed by Si treatment (62.5% and 65.78%) compared with untreated stressed plants.

It is noticeable in Figure 5B that chlorophyll b considerably declined in lettuce stressed plants; chlorophyll b significantly declined at a low level of salinity (45.36% and 42.85%) compared with the control treatment in both seasons. Additionally, chlorophyll b was significantly reduced at the high salinity level (60.82% and 55.02%) in the two seasons. Nevertheless, Si and *B. thuringiensis* led to remarkable increases in chlorophyll b. The best result (91.5% and 81.48%) was achieved with *B. thuringiensis* treatment in the stressed plants at low salinity level in comparison with the untreated stressed plants.

### 2.6. Number of Leaves, Head Weight and Total Yield

**Figure 6:**
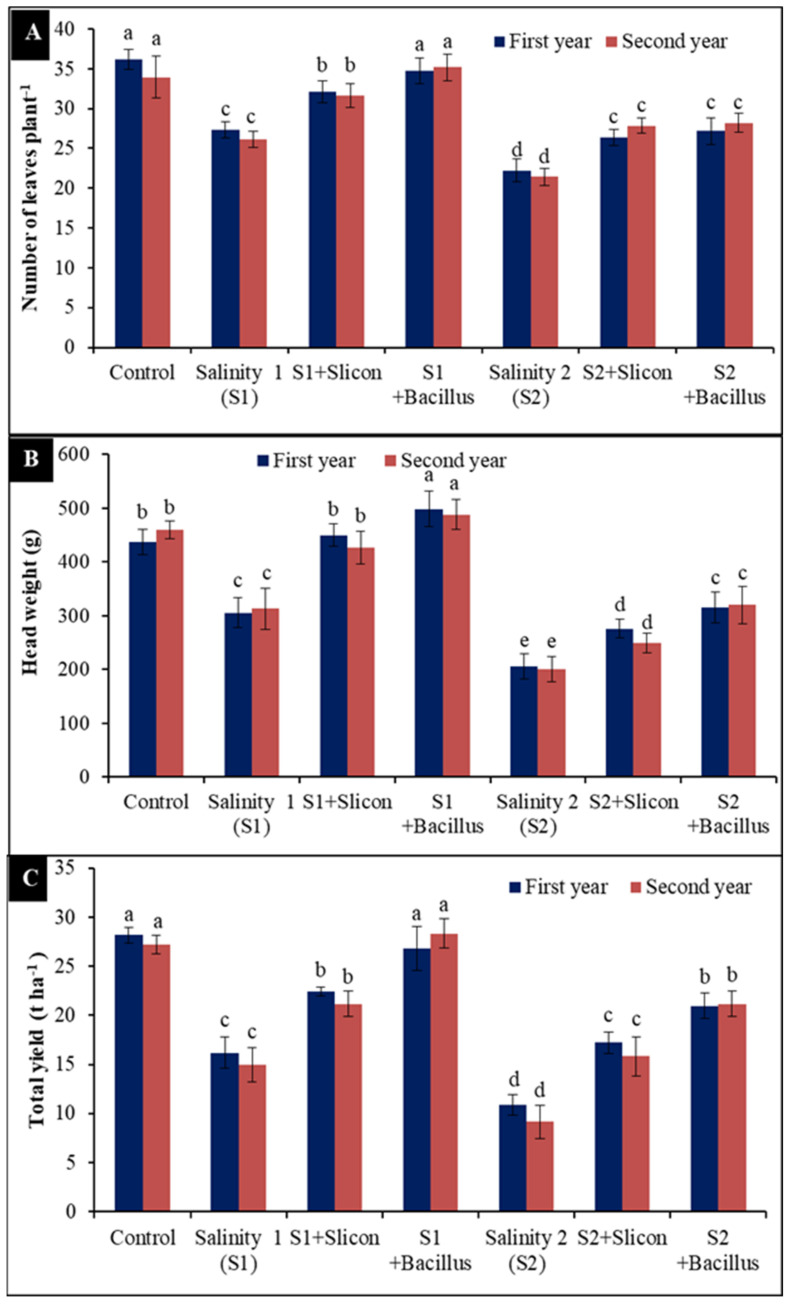
Effect of Si (potassium silicate at 2.7 mmol L^−1^) and *B. thuringiensis* on leaves number (**A**) head weight (**B**) and total yield (**C**) in lettuce plants under salinity during two seasons (2019/2020 and 2020/2021). Different letters on the columns show significant differences between the treatments according to ANOVA, Duncan’s multiple range test at 0.05 level. Data is the mean (±SE) of tree replicates. Salinity 1 (S1): 4 dS m^−1^, Salinity 2 (S2): 8 dS m^−1^.

Considerable differences were recorded in number of leaves, head weight, and total yield of lettuce plants (Figure 6A–C). Our results revealed that both salinity levels (4 dS m^−1^ and 8 dS m^−1^) caused a considerable reduction in the number of lettuce leaves in the first year (24.50% and 32.92%) and in the second year (27.73% and 35.71%) (Figure 6A). Similarly, head weight was also significantly decreased (30.02%, 52.94% and 31.87%, 56.23%) compared to the control in each of the two seasons, respectively (Figure 6B). Obtained results in Figure 6C revealed a statistically significant decrease in total yield (t ha^−1^) in stressed lettuce plants (42.55%, 62.77% and 45.11%, 66.43%) compared with the control treatment. Nevertheless, *B. thuringiensis* and Si considerably augmented number of leaves, head weight (g), and total yield in lettuce plants under saline conditions in comparison with untreated plants. Under the salinity levels, *B. thuringiensis* gave the maximum results and considerably increased number of leaves (26.6% and 26.05%), head weight (39.65% and 37.9%), and total yield (36.49% and 47.75%) compared with untreated stressed plants in both seasons.

## 3. Discussion

In the current study, we provided evidence of Si exogenous application and *B. thuringiensis* seed treatment in the mitigation of salt stress effects on lettuce plants. The exposure of lettuce plants to two different salinity levels (4 dS m^−1^ and 8 dS m^−1^) showed harmful effects on number of leaves, head weight, and total yield. This damaging effect of salinity on these characteristics might be due the impact of salinity on decreasing water and nutrients uptake from the soil, decreasing the elongation of roots, and cell division in shoots, consequently decreasing growth and physiological characteristics [6,7], resulting in the reduction in morphological characters and total yield in lettuce plants. Also, salinity may induce ion toxicities such as high Na^+^, Cl^−^, or sulfate (SO_4_^2−^) which decrease the uptake of essential elements like nitrogen, phosphorus, and potassium [52]. The increase in salinity concentration in soil may result in a reduction in water potential and affect many biochemical and physiological processes [53]. Similar results have been reported by Alomran et al. [54] and Kim et al. [55]. Contrariwise, *B. thuringiensi* and Si application led to improvements in number of leaves, head weight, and total yield in stressed lettuce plants. This positive effect of B. thuringiensi and Si could be due to the essential role of *B. thuringiensis* in mitigating the adverse effect of salinity by reducing ethylene and production of indole acetic acid by *B. thuringiensis* that not only enhanced root elongation but also increased NPK concentration in stressed plants [56]. In addition, Si application increased N and P uptake and could regulate the absorption and mobility of N in the plant, maintaining the optimum level of N and increasing N use efficiency [57], consequently improving the yield of stressed plants.

Ascorbic acid, chlorophyll a, b, and RWC decreased significantly according to the adverse effects of salinity. This unfavorable effect of salinity, mainly the high salinity level, could be due to the harmful impact of salinity on AsA and the reduction of secondary metabolites [58], decrease in energy transport from PSII to PSI [59], and adverse effect on the chloroplast structure and formation i salt stressed plants [3]. The injurious impact of salinity on chlorophyll was also due to the decrease in stomatal movements and the damage of many biological processes. These results are in harmony with those recorded by Abdelaal et al. [20] and Islam et al. [60] in stressed sweet pepper plants. Additionally, salinity adversely affects RWC, which could be due to the detrimental effect of salinity on the cell wall, increasing the synthesis of stress ethylene, which plays vital role in the loss of membrane stability and chlorophyllase activation, decreasing osmotic potential and water status, consequently decreasing RWC % [61,62]. The damaging influence of salinity on AsA, chlorophyll a, b, and RWC can be mitigated by silicon and seed treatment with *B. thuringiensis*. These results, perhaps due to Si, can play a supportive role in increasing the uptake and concentration of K^+^, as well as reducing Na+ uptake [17], which enhances enzyme activity and improves water status, photosynthesis, and RWC [63]. Similarly, the positive role of *B. thuringiensis* in increasing AsA, chlorophyll a, b, and RWC in stressed lettuce plants is perhaps due to its valuable effect on root growth and ability to increase water availability. Also, plant growth promoting rhizobacteria (PGPR) can produce exopolysaccharides which enhances the soil structure and increases the availability of soil water [64], improving physiological characters, especially chlorophyll a, b, and RWC, under stress conditions [65,66].

Salinity stress negatively influenced and significantly increased EL%, proline content and MDA. This negative impact on EL% is probably due to its injuring effect on the cell membrane and permeability process. Similar results were recorded by Abdelaal et al. [20]. Treatment of lettuce plants with Si and *B. thuringiensis* resulted in a decrease in EL%, proline content, and MDA. This synergistic impact of Si and *B. thuringiensis* treatment was probably due to the helpful roles of Si and *B. thuringiensis* in improvement of the membrane integrity and the permeability of the plasma membrane. Salinity stress has a deleterious effect on lettuce plants and increases proline content; proline was significantly augmented as a response to salinity, and this harmful influence of salinity might be due to a decrease in the oxidation of proline to glutamate, consequently increasing proline content [67]. Moreover, the application of Si and seed treatment with *B. thuringiensis* led to the adjustment of the osmotic balance and regulation of proline content in stressed lettuce plants. MDA considerably increased in lettuce plants under low salinity levels, a high increase was recorded with the high level of salinity, this increase could be due to the fact that MDA is one of the reactive compounds and a signal for many stresses, particularly salinity, and causes harmful effects to proteins, lipids, and the electron transport chain [68]. Contrariwise, the stressed lettuce plants indicated a significant reduction in MDA because of the application of SI and seed treatment with *B. thuringiensis*. This helpful effect in decreasing MDA could be due to the role of Si and *B. thuringiensis* in improving phenol content and enzyme activity, which protects proteins and lipids from oxidative stress and reduces MDA formation in the stressed lettuce plants. These results are in accordance with those recorded by Sharma et al. [69]. Also, Si application can decrease the tocopherol radical and oxidative damage in the plant cells, and consequently increase α-tocopherol, which may neutralize MDA content by decreasing reactive oxidative anions and helped to stabilize membrane integrity [70].

It is well known that antioxidant enzymes play a crucial role in plant tolerance to stress. In the present study, we found that CAT, SOD, POX, and PPO were accumulated significantly in the stressed lettuce plants, and this accumulation is an important approach that helps plants to deal with numerous stresses and as the main scavenging enzymes involved in ROS scavenging in several plants [20,34,36]. Moreover, application of SI and *B. thuringiensis* led to adjusted CAT, SOD, POX, and PPO activity in stressed lettuce plants. The significant effect of *B. thuringiensis*, perhaps due to the production of growth substances such as auxin, cytokinin, and nutrient availability, as well as the upregulation of essential enzymes [65]. Interestingly enough, Si application can activate the plant defenses by boosting the activity of enzymes CAT, SOD, POX, and PPO, whic, scavenge ROS and protect the cells from oxidative damage [71].

A very important signal under salinity stress conditions is ROS generation, which causes membrane disturbance and increases EL% and MDA. Under the two salinity levels, the excessive accumulation of O_2_^−^ and H_2_O_2_ was recorded in lettuce plants; this accumulation of reactive compounds can cause oxidative stress to lipids, proteins, and nucleic acids [65]. Also, the increase of reactive oxygen species was recorded under drought stress in sugar beet plants [72]. Nonetheless, the harmful impact of salinity was overcome by Si application and *B. thuringiensis*, which reflect a significant reduction in O_2_^−^ and H_2_O_2_ levels. Therefore, it is possible that seed treatment with *B. thuringiensis* can obstruct ROS formation by adjusting enzymatic and non-enzymatic antioxidants. Also, *B. thuringiensis* was reported to counteract the oxidative stress of ROS under saline conditions in sweet pepper and keep the cell membrane from being damaged [6]. Additionally, Si application led to mitigation of the harmful influence of salinity in lettuce plants and significantly decreased the levels of O_2_^−^ and H_2_O_2_, because Si plays a vital role as an anti-stress compound [71,73]. In summary, salinity stress caused a reduction in number of leaves, head weight, total yield of lettuce plants, and increase in EL%, MDA, and reactive oxygen species. Nonetheless, the application of Si and seed treatment with *B. thuringiensis* led to mitigation of the harmful influence of salinity by increasing the photosynthesis process and RWC, and regulating proline content and enzyme activity in stressed lettuce plants.

## 4. Materials and Methods

### 4.1. Experimental Designe and Plant Materials

The experiments were performed in pots at Kafrelsheikh Univ., Botany Dept. during 2019/2020 and 2020/2021 seasons, to examine the effect of *Bacillus thuringiensis* MH161336 106–8 CFU/cm^3^ as seed treatment and Si (potassium silicate at 2.7 mmol L^−1^) as foliar spray on lettuce under the different salinity levels (4 dS m^−1^ and 8 dS m^−1^). The biochemical and physiological characteristics were performed at the EPECRS Excellence Center and PPBL Lab., Kafrelsheikh University. The seeds of lettuce (*Lactuca sativa* L.) cv. SUSANA were arranged in three groups; one of them was treated with *B. thuringiensis* and the other two went without treatments. The seeds were surface sterilized for 5 min by sodium hypochlorite 2.5%, 70% ethanol for 1 min, and then washed with distilled water four times. *B. thuringiensis* MH161336, the final concentration of Bacterial cultures was 10^6–8^ CFU/cm^3^ [42], seeds were kept at room temperature for 6 h and then sown in trays in the nursery on the 17^th^ and 19^th^ of September in both seasons, respectively. The transplanting was done in pots 40 cm in diameter fifty six days after the sowing, each pot having two plants. The plants were divided into three collections (control, *B. thuringiensis* treatment, potassium silicate at 2.7 mmol L^−1^). Saline water with two salinity levels (4 dS m^−1^ and 8 dS m^−1^) from NaCl was used to irrigate the plants and the group with Si was treated with potassium silicate at 2.7 mmol L^−1^ twice, 15 and 30 days after transplanting. Fertilizer containing nitrogen, phosphorus, and potassium (NPK) (48:72:48 kg ha^−1^) was used in one dose before transplanting. The treatments were arranged in a completely randomized design with three replications and the samples were selected for morphological, biochemical, and physiological studies at 60 days from transplanting, while head weight and plant and total yield were calculated at 80 days from transplanting.

### 4.2. Biochemical and Physiological Characters

#### 4.2.1. Lipid Peroxidation (MDA) Determination

Lipid peroxidation was assayed as malondialdehyde (MDA) at 532 and 600 nm using spectrophotometer. MDA (μmol g^−1^ FW) = [6.45 × (A532 − A600) − (0.56 × A450)] × V^−1^W, where V = volume (cm^3^); W = weight (g) [74].

#### 4.2.2. Hydrogen Peroxide (H_2_O_2_) and Superoxide (O_2_^−^)

Lettuce leaves were infiltrated with 10 mM potassium salicylate buffer containing 0.1% (*w*/*v*) NBT or 0.1 *w*/*v* % DAB. H_2_O_2_ and O_2_^−^ were assayed as arbitrary units using the method of Huckelhoven et al. [75].

#### 4.2.3. Electrolyte Leakage Assay (EL%)

Ten fresh leaf discs (1 cm^2^) of lettuce were placed into bottles in 25 cm^3^ deionized water. Bottles were shaken for 20 h, electrical conductivity was recorded for each, and then flasks were heated (80 °C) for 1 h, and the samples were shaken again (at 21 °C) for 20 h. Final conductivity was calculated for each flask. Electrolyte leakage % was calculated with the following formula: initial/final conductivity × 100 [76].

#### 4.2.4. Enzymes Assay

Frozen lettuce leaves were used for protein extraction; the frozen leaves were ground in liquid nitrogen using ice cold mortar and pestle. Protein was extracted according to Bradford [77]. Enzyme activity was assayed in supernatant; CAT activity was assayed using spectrophotometer at 240 nm based on the rate of H_2_O_2_ consumption as μmol min^−1^ mg protein^−1^ [78]. A SOD activity was measured at 560 nm as μmol min^−1^ mg protein^−1^ according to Giannopolitis and Ries [79]. Peroxidase (POX) activity was assayed as described by Hammerschmidt et al. [80]. Polyphenol oxidase (PPO) activity was assayed according to the method described by Malik and Singh [81].

#### 4.2.5. Relative Water Content (RWC%)

Twenty fresh leaf discs of lettuce were taken to determine RWC, RWC% was calculated as follows: RWC = (FW − DW)/(TW − DW) × 100 [82].

#### 4.2.6. Determination of Proline

Proline in fresh lettuce leaves was determined using a spectrophotometer at 520 nm and calculated as μg g^−1^ FW [83].

#### 4.2.7. Determination of Ascorbic Acid

Samples of leaves were taken at the harvesting date to determine ascorbic acid (AsA) (vitamin C mg 100 g^−1^ FW) according to the Association of Official Analysis Chemists (A.O.A.C) [84].

#### 4.2.8. Chlorophyll A and B Determination

The extraction from fresh leaves was prepared using N-N Dimethyl formamide and the chlorophyll a and b was determined at 647 and 664 nm using a spectrophotometer [85].

### 4.3. Morphological and Head Yield Characters

Number of leaves, head weight, and total yield were recorded.

### 4.4. Statistical Analysis

The results were statistically analyzed using ANOVA procedures [86] using the MSTAT-C statistical software package. Duncan was used to compare the means between treatments [87] when the difference was significant (*p* ≤ 0.05).

## 5. Conclusions

Our results offer insights into the possible efficiency of *B. thuringiensis* as a seed treatment and Si (potassium silicate at 2.7 mmol L^−1^) as a foliar application in the alleviation of the deleterious impacts of salinity in lettuce plants and to increase yield production. These treatments cause increases in the number of leaves, chlorophyll a and b, and RWC, as well as total yield under the salinity levels. On the other side, the stress signals such as ROS (O_2_^−^ and H_2_O_2_), MDA, and EL% were reduced considerably in stressed lettuce plants as a positive effect of these treatments. Therefore, we recommend the application of Si as a foliar application and *B. thuringiensis* as a seed treatment to mitigate the adverse impact of salinity on lettuce plants by improving the antioxidant system and improving plant production in the agro-biological system.

## Data Availability

Data is contained within the article.
